# El Acceso Temprano a la Neurorrehabilitación es Clave Para Reducir la Duración de la Amnesia Postraumática

**DOI:** 10.31083/RN33473

**Published:** 2025-07-24

**Authors:** Alberto García-Molina, Daniela Carvallo-Fliman, Èlia Vilageliu-Jordà, Antonia Enseñat-Cantallops

**Affiliations:** ^1^Institut Guttmann, Institut Universitari de Neurorehabilitació adscrit a la UAB, 08916 Badalona, España; ^2^Fundació Institut d’Investigació en Ciències de la Salut Germans Trias i Pujol, 08916 Badalona, España; ^3^Departamento de Psicología Clínica y de la Salud, Universitat Autònoma de Barcelona, 08193 Bellaterra, España; ^4^Centro de Estudios en Neurociencia Humana y Neuropsicología, Facultad de Psicología, Universidad Diego Portales, 8370067 Santiago de Chile, Chile

Las personas que sobreviven a un traumatismo craneoencefálico (TCE) pueden presentar un trastorno parcial de la conciencia denominado amnesia postraumática (APT). La APT es un estado transitorio de confusión y perturbación global del funcionamiento cognitivo-conductual que se caracteriza por una desorientación en persona, espacio y tiempo, por la incapacidad para recordar eventos anteriores al TCE (amnesia retrógrada) y dificultad para establecer nuevos recuerdos a partir de acontecimientos posteriores a la lesión cerebral (amnesia anterógrada). Asimismo, estas personas tienen problemas para organizar sus pensamientos, mezclan información procedente de diferentes fuentes (externas o internas), y el pasado, el presente y el futuro se desdibujan, generando una realidad que los sitúa en el aquí y el ahora. Estos síntomas cognitivos acostumbran a ir acompañados por una desregulación emocional y/o conductual (p. ej., labilidad afectiva, irritabilidad, inquietud, agresividad, hipoactividad) [1, 2].

La duración de la APT es uno de los marcadores clínicos más fiables para pronosticar la capacidad funcional post-TCE: más tiempo en APT, peor pronóstico funcional [3]. En 2022 publicamos un artículo en el que analizamos que variables predecían su duración [4]. En ese momento nos centramos en analizar el comportamiento de las personas que emergían de APT durante su estancia en nuestro hospital; y excluimos del estudio a todas aquellas que al alta hospitalaria permanecían en esta fase post-TCE. Poco después de su publicación, nos asaltó una duda: si todas las personas que atendemos en el hospital realizan programas de neurorrehabilitación similares, ¿Por qué unas emergen de la APT y otras no? Este estudio tiene como objetivo tratar de esclarecer esta cuestión.

Entre enero de 2019 y octubre de 2023 atendimos a 101 personas en APT, 22 de las cuales permanecían en APT en el momento del alta hospitalaria (a este conjunto de personas lo denominamos Grupo alta-APT). Se estableció un grupo de contraste (lo denominamos Grupo emerge-APT) formado por las 22 primeras personas que emergieron de APT durante su estancia hospitalaria (es decir, fueron seleccionadas en función de su orden de llegada al centro). El estudio fue aprobado por el the Research Committee of the Institut Guttmann Neurorehabilitation Hospital and the Research Ethics Committee of the Fundació Unió Catalana Hospitals (código CEI 23/47).

Los análisis estadísticos se llevaron a cabo con el programa SPSS 29,0 (IBM Corp., Armonk, NY, USA) (con un nivel de significación *p* < 0,05). La prueba de Shapiro Wilk constató que las variables incluidas en el estudio no seguían la distribución normal. Por ello, las comparaciones intergrupales se realizaron con la prueba no paramétrica U de Mann-Whitney.

No se observaron diferencias estadísticamente significativas (*p* < 0,05) entre ambos grupos en las siguientes variables: edad en el momento del TCE (U Mann-Whitney = 170,5; *p* = 0,093), sexo (U Mann-Whitney = 209; *p* = 0,268), nivel formativo -estudios reglados cursados por la persona- (U Mann-Whitney = 194,5; *p* = 0,229), gravedad del TCE -según la puntuación en la *Glasgow Coma Scale*- (U Mann-Whitney = 167,5; *p* = 0,181) y presencia de lesiones axonales difusas grado III (U Mann-Whitney = 224; *p* = 0,837). Hallamos diferencias estadísticamente significativas en la puntuación de la primera administración de la *Galveston Orientation and Amnesia Test* (GOAT) realizada en nuestro hospital (U Mann-Whitney = 147; *p* = 0,025). Las puntuaciones del Grupo alta-APT eran menores que las del Grupo emerge-APT (mediana: 15; rango intercuartílico (IQR): 22 vs. mediana: 42,5; IQR: 36). Asimismo, encontramos diferencias en el tiempo transcurrido entre el TCE y el ingreso en nuestro hospital (U Mann-Whitney = 111,5; *p* = 0,002). El Grupo alta-APT fue admitido 94,38 días (IQR: 71) después del TCE, mientras que el Grupo emerge-APT 54,55 días (IQR: 43). A tenor de la diferencia intergrupal en esta última variable, y sin subestimar las discrepancias en la GOAT, creemos que es pertinente destacar la importante del tiempo que transcurre entre el momento en el que se produce la lesión y el inicio del proceso neurorrehabilitador.

La guía “Principios básicos de la neurorrehabilitación del paciente con daño cerebral adquirido”, redactada por la Sociedad Española de Neurorrehabilitación, advierte que el proceso rehabilitador post-TCE debe iniciarse tan pronto como sea posible (siempre y cuando no haya complicaciones médicas que pueden comprometer el estado vital del paciente) [5]. Nuestros resultados ratifican esta postura, mostrando que el retraso en el acceso al tratamiento influye en la duración de la APT. Las personas que fueron dadas de alta en APT iniciaron la rehabilitación, de media, 40 días más tarde que aquellas que emergieron de APT durante su ingreso hospitalario. Esta demora podría explicarse por diversos motivos (p. ej., estado médico de la persona, dilación de los procesos administrativos, disponibilidad de camas en el centro neurorrehabilitador, incidencias inespecíficas…). Desafortunadamente, no hemos tenido acceso a esta información.

En conclusión, el tiempo que transcurre entre el TCE y el inicio de la neurorrehabilitación puede condicionar la duración de la APT. De tal forma, es necesario que los sistemas de derivación sanitarios actúen de forma coordinada para agilizar el acceso a centros especializados con el objetivo de maximizar los potenciales efectos terapéuticos de la neurorrehabilitación.

## Data Availability

En caso de interés por los datos reportados en este trabajo contactar con el autor de correspondencia.

## Contribuciones de los Autores

AGM diseñó el estudio de investigación. DCF y EVJ llevaron a cabo la investigación. AGM y DCF analizaron los datos. AGM, DCF y EVJ redactaron los borradores. AEC brindó apoyo y asesoramiento para el estudio. Todos los autores contribuyeron a los cambios editoriales del manuscrito. Todos los autores leyeron y aprobaron el manuscrito final. Todos los autores han participado lo suficiente en el trabajo y han acordado ser responsables de todos los aspectos del mismo.

## Aprobación Ética y Consentimiento Informado

El estudio se llevó a cabo de acuerdo con las directrices de la Declaración de Helsinki y fue aprobado por el Comité de Investigación del Hospital de Neurorrehabilitación Institut Guttmann y el Comité de Ética de Investigación de los Hospitales de la Fundació Unió Catalana (código CEI 23/47). Dada la naturaleza retrospectiva del estudio, y la imposibilidad de recoger el consentimiento informado de los participantes, se pide al CEI de la Fundació Unió la exención del requisito de su recogida.

## Agradecimientos

Gracias a Institut Guttmann por la posibilidad de realizar el trabajo.

## Financiación

Esta investigación no recibió financiación externa.

## References

[1] Parker TD, Rees R, Rajagopal S, Griffin C, Goodliffe L, Dilley M (2022). Post-traumatic amnesia. *Practical Neurology*.

[2] Ponsford J, Trevena-Peters J, Janzen S, Harnett A, Marshall S, Patsakos E (2023). INCOG 2.0 Guidelines for Cognitive Rehabilitation Following Traumatic Brain Injury, Part I: Posttraumatic Amnesia. *The Journal of Head Trauma Rehabilitation*.

[3] Walker WC, Stromberg KA, Marwitz JH, Sima AP, Agyemang AA, Graham KM (2018). Predicting Long-Term Global Outcome after Traumatic Brain Injury: Development of a Practical Prognostic Tool Using the Traumatic Brain Injury Model Systems National Database. *Journal of Neurotrauma*.

[4] García-Molina A, Andreu-Tello A, Vilageliu-Jordà E, Enseñat-Cantallops A (2022). Predictors of the duration of post-traumatic amnesia following traumatic brain injury. *Revista De Neurologia*.

[5] Noé E, Gómez A, Bernabeu M, Quemada I, Rodríguez R, Pérez T (2021). Guía: Principios básicos de la neurorrehabilitación del paciente con daño cerebral adquirido. *Recomendaciones de la Sociedad Española de Neurorrehabilitación*.

